# Enhanced Nitric Oxide Detection Performance of Layer-like Ni-Doped WO_3_-Based Photoinduced Gas Sensor at Room Temperature

**DOI:** 10.3390/ma19132771

**Published:** 2026-06-30

**Authors:** Na Fang, Shaoling Wang, Leilei Zhang, Xianju Shi, Haoran Ma, Jichao Wang

**Affiliations:** 1 Puyang Institute of Technology, Henan University, Puyang 457000, China; pyfangna@126.com; 2 Henan Key Laboratory of Nanocomposites and Applications, Institute of Nanostructured Functional Materials, Huanghe Science and Technology College, Zhengzhou 450006, China; luanzhanglei123@163.com; 3 College of Chemistry and Chemical Engineering, Henan Institute of Science and Technology, Xinxiang 453003, China; mhrmhaoran@163.com

**Keywords:** WO_3_, photoinduced gas sensor, Ni doping, NO detection, room temperature

## Abstract

**Highlights:**

- Photoinduced WO_3_-based sensor exhibited good selectivity and stability for NO gas with ppb level.
- Doped Ni accelerated the electron transfer to form superoxide radicals.
- Layer-like Ni-doped WO_3_ was synthesized through the spraying-calcination method.

**Abstract:**

Nitric oxide (NO) detection at low concentrations was of significant practical importance for the screening and monitoring of certain respiratory diseases, driving the demand for gas sensors with enhanced performance and reduced power consumption. This study presented a photoinduced NO gas sensor based on layer-like Ni-doped WO_3_ nanomaterials operating at room temperature (RT). The synergistic effect of Ni doping and photoactivation enabled remarkable gas sensitivity across a low concentration range (10~100 ppb), achieving rapid response/recovery times (28 s/50 s) at 50 ppb under RT. The limit of detection (LOD) for NO molecule could reach below 8.29 ppb. A good linear correlation between the response value and NO concentration was demonstrated under a wide relative humidity range (20~90%). Ni doping induced oxygen vacancies while simultaneously facilitating photoinduced electron transfer for surface oxygen activation. The optimized sensor maintained good response stability after three months of ambient storage, demonstrating excellent operational durability. In situ experimental results further elucidated that the doped Ni site enhanced electron transfer from the surface to adsorbed oxygen molecules, generating superoxide radicals. This work provided fundamental insights into surface engineering strategies for developing optically modulated gas sensors and proposed a viable pathway for constructing energy-efficient exhalation monitoring systems.

## 1. Introduction

With the rapid development of industry and society, human health monitoring and disease screening have gradually attracted attention [1,2]. Since exhaled breath contains gas molecules characteristic of various diseases, breath analysis has become an ideal non-invasive measurement [3]. For example, the NO molecule was a crucial indicator of some respiratory diseases [1]. However, the concentration of exhaled NO was typically on the order of several tens of ppb [1,3]. Due to its low concentration, traditional detection techniques often require expensive equipment, such as gas chromatography (GC) and liquid chromatography [4]. Recently, the development of low-cost gas sensors has increasingly focused on medical diagnosis [3,5]. Yet, the selectivity, stability, and sensitivity (3S) of gas sensors are mainly influenced by the sensing materials [1,3,5]. In contrast, owing to their low-cost fabrication, material stability, and scalable manufacturing processes, semiconductor-based chemiresistive sensors have garnered significant research interest.

WO_3_ material, composed of W-O octahedra connected by shared oxygen atoms, exhibited an alterable surface structure [6,7]. A series of WO_3_-based materials have demonstrated excellent gas-sensitive properties for NO, NO_2_, C_2_H_2_, H_2_S, and acetone molecules [6,8,9,10,11]. To further promote their performance, various methods, including metal loading, heterojunction construction, and organic surface modification, have been employed to enhance the content of active electrons on the surface, leading to improved sensitivity towards low-concentration gas molecules [9,12]. In particular, ion doping not only improves electron transfer at the interface but also introduces oxygen defects on the surface, thereby reducing the activation energy barrier for adsorbed oxygen molecules [10]. Y. Xia et al. synthesized Ta-doped WO_3_ materials with enriched oxygen vacancies, achieving excellent NO discrimination at ultralow concentrations of 50 ppb [13]. Variable valence transition metal doping, such as with Ni, Fe, and Co, has consistently played a key role in electron transfer on semiconductor surfaces in electrocatalytic and photocatalytic processes [14]. J.M. Song proved that Ni doping promoted electron transfer on the W_18_O_49_ surface, reducing the response–recovery time of an n-butanol gas sensor [10]. Ni-doped WO_3_-based gas sensors have achieved the best target gas selectivity for 2 ppm acetone at RT [14]. Additionally, Ni doping not only constructs electron transfer channels on the surface but also introduces oxygen vacancies in WO_3_, thereby adjusting the surface adsorption capacity and selectivity. The design of Ni-doped WO_3_ materials could potentially contribute to highly sensitive gas sensors for low-concentration NO detection.

Furthermore, the excellent light absorption capacity of WO_3_ should not be overlooked, as it can serve as an effective alternative to thermal activation, promoting the activation of adsorbed molecules and generating more photoinduced active electrons for gas sensing of ppb-level NO [15,16]. In the context of the high-humidity testing environment associated with NO detection in exhaled air, light illumination could reduce the operating temperature of the gas sensor, thereby extending the device’s service life [12,15,16,17]. J.W. Yoon et al. reported that Zn-doped WO_3_ materials achieved reliable ppb-level NO_2_ detection under specific visible-light activation [18]. Therefore, the synergistic combination of defect-engineered WO_3_ nanostructures and photoactivation offered a promising strategy for developing an energy-efficient NO sensor operating at room temperature.

In this regard, a series of layer-like Ni-doped WO_3_ (Ni-WO_3_) materials were synthesized and employed as sensing materials for NO detection under blue LED light illumination. The photoactivated Ni-WO_3_ exhibited significantly enhanced NO-sensitive performance at room temperature, with a low concentration range, from 10 ppb to 100 ppb. Such excellent gas sensitivity was attributed to the combined effects of Ni doping and light illumination. The linear correlation between the response value and NO concentration of the photoinduced gas sensor was investigated under different humidity and subsequently compared with that of GC measurements. The influence of Ni doping on the surface structure was characterized by ESR and TEM. Moreover, through in situ X-ray photoelectron spectroscopy (XPS) and electron spin resonance (ESR), the possible mechanism for NO detection was inferred and subsequently confirmed.

## 2. Materials and Methods

The Ni-doped WO_3_ sample (Ni-WO_3_) was synthesized via a spraying-calcination method. Ammonium metatungstate hydrate and nickel(II) nitrate hexahydrate (Shanghai Macklin Biochemical Technology Co., Ltd., Shanghai, China) were adopted as W and Ni sources. The precursor powder was obtained from spraying method with the above mixed solution as the spray solution. The final sample was attained after calculation and denoted as xNi-WO_3_, where x represented the ideal mol/mol % ratio between Ni and W atoms. The morphology and composition of the obtained samples were characterized using XRD (D8 Advance, Bruker, Karlsruhe, Germany), XPS (Escalab 250XI^+^, Thermo Fisher Scientific, Waltham, MA, USA), TEM (Tecnai G^2^ F20 S-TWIN, FEI, Hillsboro, OR, USA), and SEM (Quanta250 FEG, FEI, USA), and their electron transfer properties were investigated by DRS (Cary-5000 UV-Vis-NIR, Agilent, Santa Clara, CA, USA), photoinduced curve (CHI660E, Shanghai CH Instruments Ins., Shanghai, China), ESR (EPR200-plus, CIQTEK, Hefei, China), EIS (CHI660E, Shanghai CH Instruments Ins., China), and in situ XPS (Escalab 250XI^+^, Thermo Fisher Scientific, USA) measurements. The NO gas-sensitive property was measured using reconstructive gas-sensitive equipment (Figure S1). The experimental and testing processes were detailed into Supplementary Materials S1–S3.

## 3. Results

**Figure 1:**
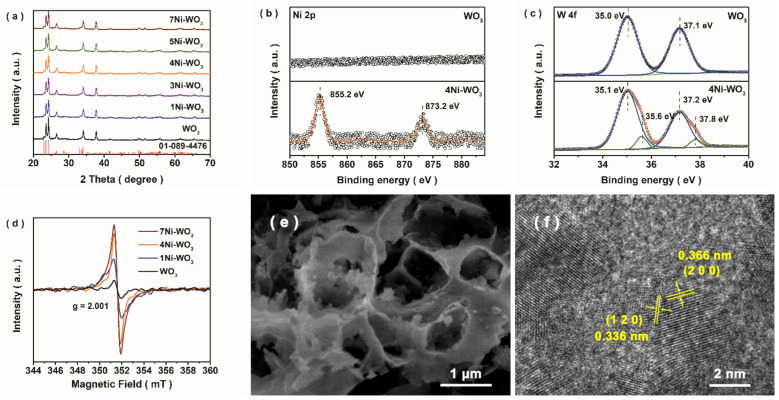
XRD pattern (**a**), XPS spectra of (**b**) Ni 2p and (**c**), W 4f (blue/green curve: W(VI)/W(V)), ESR spectrum (**d**), SEM (**e**), and TEM (**f**) of the obtained samples.

As shown in Figure 1a, the observed diffraction peaks of all obtained samples corresponded to the characteristic peaks of monoclinic phase WO_3_. No additional diffraction peaks were detected upon Ni doping. Compared with the standard card (PDF No. 01-089-4476), the intensity of the (0 0 2) characteristic peak at 23.0° was weaker, while that of the (1 3 0) characteristic peak at 37.7° was stronger, indicating anisotropic growth with inhibition along the [0 0 1] direction. This may lead to the formation of layer-like WO_3_ with a higher surface area. As summarized in Table S1, the Ni content in the doped WO_3_ samples gradually increased with the designed Ni/W molar ratio during synthesis. In Figure 1b, only two strong peaks at 855.2 eV and 873.2 eV were observed for the 4Ni-WO_3_ sample, corresponding to the characteristic peaks of Ni(II) 2p_1/2_ and 2p_3/2_, respectively [10]. As shown in Figure 1c, apart from two strong peaks of W(VI), two weak peaks at 35.6 eV and 37.8 eV appeared, indicating the generation of W(V) induced by Ni doping [19]. Meanwhile, the ESR results (Figure 1d and Figure S2) showed that the signal intensity changed, suggesting that oxygen vacancy was generated and increased with increasing Ni doping content [20]. In Figure 1e and Figure S3, the 4Ni-WO_3_ samples exhibited a layer-like morphology, and the average thickness could be calculated to be about 6.8 nm. Furthermore, clear lattice fringes with spacings of 0.366 and 0.336 nm were observed in the TEM image (Figure 1f), corresponding to the (2 0 0) and (1 2 0) crystal planes of monoclinic WO_3_, respectively. Based on the above results and analysis, Ni-doped WO_3_ layers with oxygen vacancy were successfully synthesized via the spraying-calcination method.

**Figure 2:**
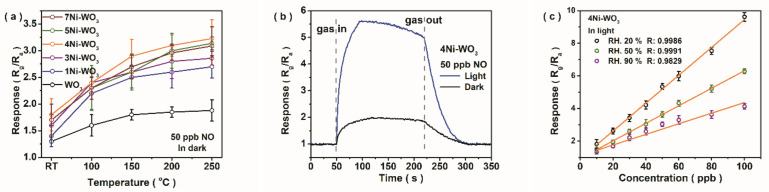

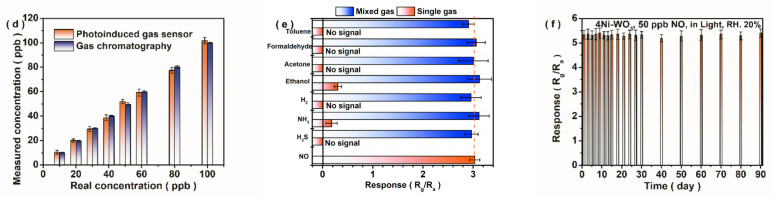
Gas-sensitive performance of the obtained Ni-WO_3_ sample. (**a**) Operating temperature, (**b**) response curve in light/dark, (**c**) linear correlation with different concentration and relative humidity, (**d**) comparison test between sensor and GC, (**e**) gas-sensitive selectivity in 90 RH and 50 ppb NO gas, and (**f**) cycling stability of 3 months.

The NO gas-sensitive performance of the obtained Ni-WO_3_ samples was investigated. As shown in Figure 2a, the response of the NO gas sensor increased with the operating temperature, and the 4Ni-WO_3_ sample exhibited the highest performance among all Ni-WO_3_ samples. In Figure 2b, the response value for NO gas at RT was significantly enhanced under blue LED light illumination. Meanwhile, the response and recovery times were all markedly shortened from 39 s/73 s to 28 s/50 s, attributed to the photoinduced generation of abundant active electrons on the WO_3_ surface. As shown in Figure 2c, a good linear correlation between the response value and NO concentration (10~100 ppb) was observed, although the response intensity decreased as the relative humidity (R.H.) increased from 20% to 90%. The LOD value for NO molecules could reach 1.75 ppb, 2.79 ppb and 8.29 ppb, respectively. Furthermore, the NO content measured by the photoinduced gas sensor was in basic agreement with that obtained by gas chromatography (Figure 2d), indicating its practicality for NO gas-sensitive applications. When exposed to other target gas molecules commonly present in breath testing (Figure 2e), the 4Ni-WO_3_ gas sensor still exhibited excellent selectivity. Even for simulated exhaled breath (Figure S4), the optimized gas sensor still showed the good gas-sensing for ppb-level NO detection. In a 3-month cycling experiment (Figure 2f), the stability of the photoinduced gas sensor was demonstrated. In conclusion, the photoinduced gas sensor assembled with 4Ni-WO_3_ materials can be effectively used for detecting NO in exhaled breath.

**Figure 3:**
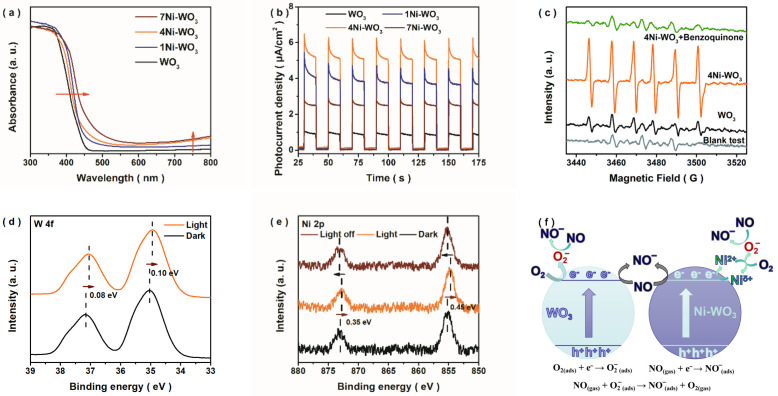
Light absorbed property (**a**), photoinduced curve (**b**), ESR spectra (**c**), in situ XPS ((**d**) W 4f and (**e**) Ni 2p), and schematic diagram (**f**) of the obtained Ni-doped WO_3_ sample.

To explore the enhanced sensitivity performance of the photoinduced gas sensor, the band structure and electron transfer behavior of the Ni-doped WO_3_ samples were systematically investigated. As shown in Figure 3a, the Ni-WO_3_ samples exhibited a broader light-response range compared to the undoped WO_3_. Notably, the light absorption ability of the 4Ni-WO_3_ and 7Ni-WO_3_ samples in the 550~800 nm range was stronger than that of undoped WO_3_ and 1Ni-WO_3_, due to the generation of oxygen vacancies. As shown in Figure 3b, a photoinduced current curve was generated under light illumination and rapidly weakened after the light was turned off. The intensity of this curve was stronger for the 4Ni-WO_3_ sample than for the others, indicating that more active photoinduced electrons could be generated within the WO_3_ materials through Ni doping and oxygen vacancies [21,22]. However, excessive oxygen vacancies led to the recombination of photoinduced electrons and holes, resulting in the decreased performance of the 7Ni-WO_3_ sample. As shown in Figure S5, the impedance spectroscopy (EIS) proved that the doped Ni site could facilitate the charge transfer of WO_3_ materials. In Figure S6, the ability for O_2_ adsorption of Ni-WO_3_ was obviously higher than in undoped WO_3_. Especially for the WO_3_-O sample, the generation of oxygen vacancy played a positive role for O_2_ adsorption in the WO_3_ surface. In addition to sufficient active electrons, the influence of the superoxide radical (O_2_^−^) on NO gas sensing could not be ignored. As shown in Figure 3c, the characteristic signal of O_2_^−^ over 4Ni-WO_3_ was much stronger than that over WO_3_, suggesting that more O_2_^−^ radicals could be generated from adsorbed oxygen molecules on the Ni-doped WO_3_ surface [22,23]. To further explore the effect of doped Ni sites on electron transfer, in situ XPS measurements were conducted, and the results are shown in Figure 3d,e. No prominent difference in the W 4f characteristic peaks was observed between light and dark conditions. Notably, the two peak positions of Ni 2p shifted toward lower energy under illumination and returned to their original positions after the light was turned off, indicating that photoinduced electrons accumulated around the doped Ni sites under illumination and that these sites could revert to their original state [21,24]. Combined with the ESR result showing an increase in O_2_^−^ radicals under light, it was inferred that doped Ni sites with variable charge states could transfer electrons from the WO_3_ surface to adsorbed oxygen molecules, thereby enhancing gas-sensitive performance. In other words, Ni doping not only introduced oxygen defects to promote oxygen adsorption ability but also constructed a fast electron transfer channel between the surface and adsorbed molecules. The proposed mechanism for NO gas sensing is illustrated in Figure 3f.

## 4. Conclusions

A series of Ni-doped WO_3_ materials were synthesized via the spraying-calcination method. The optimal 4Ni-WO_3_ sample exhibited improved NO gas-sensitive performance. Notably, the photoinduced gas sensor based on the Ni-WO_3_ sample demonstrated a linear response in the NO concentration range of 10 to 100 ppb under varying humidity levels (20%~90%). Meanwhile, its response and recovery time under light illumination was significantly shortened from 39 s/73 s in the dark to 28 s/50 s, and the LOD value for the NO molecules could reach below 8.29 ppb at 20~90% R.H. The sensor also showed good selectivity towards various exhaled target molecules. The Ni-doped WO_3_ photoinduced gas sensor exhibited NO detection capabilities comparable to those of GC. ESR and in situ XPS results confirmed that Ni doping not only generated abundant photoinduced active electrons but also constructed a fast electron transfer channel between the surface and adsorbed molecules. The findings of this study may provide insights into the design of photoinduced gas sensors for NO detection in breath testing.

## Data Availability

The original contributions presented in this study are included in the article/Supplementary Material. Further inquiries can be directed to the corresponding authors.

## Supplementary Materials

The following supporting information can be downloaded at: https://www.mdpi.com/article/10.3390/ma19132771/s1, S1. Sample characterization. S2. Gas sensing characterization. S3. Gas sensing characterization. Figure S1. Schematic diagram of testing system for NO-gas-sensitivity performance. Figure S2. EPR spectra of 4Ni-WO_3_, WO_3_ and WO_3_-O samples. Figure S3. TEM image of 4Ni-WO_3_ sample. Figure S4. Gas-sensing performance of 4Ni-WO_3_ gas sensor for NO detection in simulated exhaled breath. Figure S5. EIS Nyquist plots of WO_3_ and 4Ni-WO_3_ sample. Figure S6. O_2_ adsorption curve of 4Ni-WO_3_, WO_3_ and WO_3_-O samples. Table S1. Element content of obtained doped WO_3_ sample by XPS measurement. Table S2. Gas sensing performance of WO_3_-based sensor for NO or NO_2_ detection in previous research and studies. References [25,26,27,28,29,30,31,32] are cited in the supplementary materials.

## References

[1] Zhao J., Xu Y., Ye Z., Zhang Q., Zhang Y., Xu Z., Zhang C., Peng N., Ren H., Li P. (2026). Heterostructure aerogels with a hole-rich medium enable portable, sub-ppb FeNO detection toward home respiratory care. ACS Sens..

[2] Singh S., Saggu I.S., Chen K., Xuan Z., Swihart M.T., Sharma S. (2022). Humidity-tolerant room-temperature selective dual sensing and discrimination of NH_3_ and NO using a WS_2_/MWCNT composite. ACS Appl. Mater. Interfaces.

[3] Jiang H., Zhang J., Li W. (2025). Ultrasensitive nitric oxide gas sensor based on gold/tin oxide composite nanofibers prepared by electrospinning. Sens. Actuators B.

[4] Yin P., You X., Cui X., Tang Z., Yu S., Fu H., Song F., Zhang K., Zhao X., Wang L. (2025). Clinically diagnose asthma and monitor its severity using an ultrasensitive chemiresistive nitric oxide (NO) gas sensor via exhaled breath analysis assisted by pattern recognition. ACS Sens..

[5] Zhu R., Gao J., Tian Q., Li M., Gao Q., Wu X., Xu S., Zhang Y. (2025). Optical chemical gas sensor based on spectral autocorrelation: A method for online detection of nitric oxide and ammonia in exhaled breath. Sens. Actuators B.

[6] Zhang J., Gao H., Leng D., Liu J., Li T., Pan C., Yu B., Zhang X., Guo J., Yang Z. (2026). Atomic-level tuning of mass-selected Ag clusters for surface functionalization of porous WO_3_ nanofibers to enhance NO_2_ sensing properties. Chem. Eng. J..

[7] Kumar A., Mazumder J.T., Joyen K., Favier F., Mirzaei A., Kim J.-Y., Kwoka M., Bechelany M., Jha R.K., Kumar M. (2025). Defect engineering approaches for metal oxide semiconductor-based chemiresistive gas sensing. Coord. Chem. Rev..

[8] Hossain M., Chatterjee K., Mohanty K., Tarafder K., Sen S., Kundu S., Varrla E., Satpati B., Pal P. (2026). Unconventional gas sensing mechanism in phase-separated n-type mixed tungsten oxide 2D-nanosheets compared against Tungsten (VI) oxide. Small.

[9] Zhang Y., Hu W., Dai J., Zhou T., Wu C., Yang M. (2026). Phase-stabilized hexagonal WO_3_ nanotube arrays with Pd-Pt dual-catalysts for high-performance fiber optic hydrogen sensing. Sens. Actuators B.

[10] Wu R., Guo S.-Q., Li Y.-C., Qi M.-Y., Ge B.-H., Song J.-M. (2024). Improving the sensing performance of rambutan-like W_18_O_49_ based gas sensor for n-butanol by Ni doping. Sens. Actuators B.

[11] Wang Y., Li J., Zhou T., Sun M., Chen S., Sun M. (2026). Modulation law theory for WO_3_-based semiconductor gas sensors detecting H_2_S gas. Chem. Eng. J..

[12] Ying Z., Zhong Y., Liu Y., Yan W. (2026). Construction of WO_3_/InWO_4_ heterojunction for NO_2_ detection at room temperature under UV activation. Anal. Chim. Acta.

[13] Chen R., Si R., Dai Q., Yang L., Hou M., He S., Li X., Zhang S., Xiang L., Guo S. (2026). Oxygen vacancy-engineered WO_3_ sensors with chemoselective gating for ultralow NO breathomics: Towards point-of-care asthma diagnostics. Chem. Eng. J..

[14] Pi M., Zheng L., Luo H., Duan S., Li C., Yang J., Zhang D., Chen S. (2021). Improved acetone gas sensing performance based on optimization of a transition metal doped WO_3_ system at room temperature. J. Phys. D Appl. Phys..

[15] Lee J., Kim M., Park S., Ahn J., Kim I.-D. (2025). Materials engineering for light-activated gas sensors: Insights, advances, and future perspectives. Adv. Mater..

[16] Wang S., Shi X., Fang N., Ma H., Wang J. (2025). Dual effects of Ag doping and S vacancies on H_2_ detection using SnS_2_-based photo-induced gas sensor at room temperature. Materials.

[17] Ling J., Zhang T., Li D., Zhang H., Tong Y., Jin L., Ji X., Zhang K., Zhang M. (2025). Constructing S-scheme charge migration in a self-assembled In_2_O_3_/WO_3_ heterojunction for photothermal-driven dual-gas detection. ACS Sens..

[18] Do N.T.T., Yu B.-H., Lee S.-W., Yu Y.-T., Yoon J.-W. (2026). Atypical humidity-enhanced NO_2_ sensing in WO_3_ via Zn doping and visible-light activation: Mechanistic elucidation and application to breath analysis. Chem. Eng. J..

[19] Wang J.-C., Shi W., Sun X.-Q., Wu F.-Y., Li Y., Hou Y. (2020). Enhanced photo-assisted acetone gas sensor and efficient photocatalytic degradation using Fe-doped hexagonal and monoclinic WO_3_ phase-junction. Nanomaterials.

[20] Abdullah S.A., Sahdan M.Z., Nayan N., Embong Z., Hak C.R.C., Adriyanto F. (2020). Neutron beam interaction with rutile TiO_2_ single crystal (1 1 1): Raman and XPS study on Ti^3+^-oxygen vacancy formation. Mater. Lett..

[21] Wang L., Cheng B., Zhang L., Yu J. (2021). In situ irradiated XPS investigation on S-scheme TiO_2_@ZnIn_2_S_4_ photocatalyst for efficient photocatalytic CO_2_ reduction. Small.

[22] Wang J.-C., Ma H., Shi W., Li W., Zhang Z., Hou Y., Zhang W., Chen J. (2024). Designed synthesized step-scheme heterojunction of Bi_2_WO_6_ nanosheet supported on CuBi_2_O_4_ nanorod with remarkable photo-assisted gas sensing for N-butyl alcohol. J. Environ. Chem. Eng..

[23] Sun T., Wang R., Guan R., Wang L., Zhong T., Liu C., Cheng X., Fang Q. (2026). Design strategies and application progress of covalent organic frameworks in photocatalytic oxidation reactions. Chem. Sci..

[24] Shi W., Zhang L., Zhang R., Yang B., Guo J., Guo X., Ma H., Wang J.-C. (2026). Construction of S-scheme CuBi_2_O_4_/BiOI heterojunction with oxygen vacancy for efficient photocatalytic CO_2_-to-CH_4_ conversion. Chin. J. Struct. Chem..

[25] Dhall S., Prakash J., Nigam A., Astakala A., Sood K. (2025). WO_3_-based Chemiresistive sensors for NO detection at low temperatures. Microchem. J..

[26] Su P.G., Li M.C. (2021). Recognition of binary mixture of NO_2_ and NO gases using a chemiresistive sensors array combined with principal component analysis. Sens. Actuators A Phys..

[27] Cai Z.X., Li H.Y., Ding J.C., Guo X. (2017). Hierarchical flowerlike WO_3_ nanostructures assembled by porous nanoflakes for enhanced NO gassensing. Sens. Actuators B Chem..

[28] Yu S., Liu X., Wang J., Li Q., Pang Y., Zhang L., Yang C., Meng Q., Wang C., Jing Q. (2026). A Room-temperature, high-ppb-level NO gas sensor based on Pt/WO_3_ co-decorated carbon nanofibers towards asthma-relevant breath analysis application. Sensors.

[29] Wu Z.-J., Chen G.-L., Li Q.-S., Lv W.-C., Xin J.-J., Song K. (2026). WO_3_ microtubules supported by nanoparticles to construct high response and low-temperature nitric oxide sensor from discarded cotton towels. Microchim. Acta.

[30] Bai H., Guo R., Zhou Y., Feng C., Chen Y., Zhang S., Feng Y., Liu W., Liu K., Guo F. (2026). A room-temperature wireless NO_2_ gas sensor enabled by WO_3_ modified V_2_CT_x_ nanosheets. Chem. Eng. J..

[31] Singh S., Gurawal P., Malik G., Adalati R., Kaur D., Chandra R. (2024). Highly responsive and selective NO gas sensing based on room temperature sputtered nanocrystalline WO_3_/Si thin films. Micro Nanostruct..

[32] Singh S., Adalati R., Gurawal P., Devi R., Malik G., Kaur D., Chandra R. (2026). Nano-interfaced WxMoyO3 heterostructure based highly selective and sensitive NO gas sensor. Sens. Actuators B.

