# Supplementary material for: Enhanced Nitric Oxide Detection Performance of Layer-like Ni-Doped WO3-Based Photoinduced Gas Sensor at Room Temperature
Source: Materials (Basel). 2026 Jun 30;19(13):2771. doi: 10.3390/ma19132771 (PMC13363474; doi:10.3390/ma19132771)
Supplement: Supplementary file 1 [file materials-19-02771-s001.zip › materials-4382177-supplementary.pdf]

Supplementary Materials

Enhanced Nitric Oxide Detection Performance of Layer-like Ni-Doped WO_3-Based Photoinduced Gas Sensor at Room Temperature

Na Fang [1], Shaoling Wang [1,*], Leilei Zhang [2], Xianju Shi [1,*], Haoran Ma [3] and Jichao Wang [3,*]

[1] Puyang Institute of Technology, Henan University, Puyang 457000, China; pyfangna@126.com
[2] Henan Key Laboratory of Nanocomposites and Applications, Institute of Nanostructured Functional Materials, Huanghe Science and Technology College, Zhengzhou 450006, China; luanzhanglei123@163.com
[3] College of Chemistry and Chemical Engineering, Henan Institute of Science and Technology, Xinxiang 453003, China; mhrmhaoran@163.com
* Correspondence: shaolingwang_518@sohu.com (S.W.); pyshixianju@163.com (X.S.); wangjichao2016@163.com (J.W.)

S1. Synthesis of Ni doped WO_3

The Ni-doped WO_3 sample (Ni-WO_3) was synthesized via a spraying-calcination method. In a typical experiment, 12.31 g of ammonium metatungstate hydrate powder was dissolved in an acid solution (pH ~6, 100 mL), followed by the addition of 0.58 g of $Ni(NO_3)_2{\cdot}6H_2O$ powder. After ultrasonic processing and stirring for 30 min, the resulting clear solution was used as the spray liquid. Subsequently, a quartz glass plate (15 cm × 15 cm) was placed on a heating pad at a temperature of 300 °C, and high-purity N_2 gas served as the carrier gas. During the spraying process, the air pressure of carrier gas was kept as about 0.05 MPa and there has approximately 10 cm space between sprayer and glass plate. The quartz glass plate loaded with the powder precursor was then immersed in ethyl alcohol solution (50 mL), and the yellow powder was obtained through ultrasonic processing, water washing, and centrifugation. After further heating at 450 °C for 2 h, the final powder was obtained and denoted as xNi-WO_3, where x represented the ideal mol/mol % ratio between Ni and W atoms. The WO_3-O sample was further synthesized through the above similar method with calcination in tube furnace with about 1.0 L/h air-flow.

S2. Characteristic Details of Measurements

The crystal structure of the synthesized photocatalysts was investigated by the powder X-ray diffraction (XRD, D8 Advance, Bruker, Germany) using Cu Kα radiation at 40 kV and 40 mA. The morphology differences of the synthesized photocatalysts were examined by field emission scanning electron microscope (SEM, FEI Quanta250 FEG USA) at an accelerating voltage of 5.0 kV. The high-resolution transmission electron microscopy was performed on a transmission electron microscope (TEM, Tecnai G^2 F20 S-TWIN, FEI, USA) operated at 200 kV. The X-ray photo-electron spectra of the as-prepared samples were analyzed using an Escalab $250XI^+$ photo-electron spectrometer with Al Ka radiation, and all the XPS data were calibrated by C 1 s standard peak at 284.8 eV. The optical

absorption and bandgap energy of the samples were characterized by ultraviolet–visible diffuse reflectance spectroscopy (UV-Vis DRS) on a Cary-5000 UV-Vis-NIR spectrophotometer with $BaSO_4$ as reference substance. Electron paramagnetic resonance (EPR) measurements were performed using an EPR200-plus spectrometer. The microwave frequency (v) was 9.83 GHz, and the modulation amplitude was 0.1 mT. The g value was calculated according to the following equation (Eq-S1):

$$g = (hv) / (\beta B_0) \qquad \text{(Eq-S1)}$$

where h, β and B_0 were on behalf of Planck constant (6.626×10^{-34} J·s), Bohr magnetron (9.274×10^{-24} J/T) and resonant magnetic field intensity (approximately 351 mT, in this study), respectively.

S3. Gas sensing characterization

The gas sensing properties of WO_3 based materials were characterized on a computer-controlled modified gas sensor test system (Fig. S1). The gas sensors were made of the alumina tube, on which two Au electrodes and platinum wires had been installed at each end.

The sensors were fabricated as follows: about15 mg of the WO_3-based samples were mixed with 2 drops of terpineol to form a paste, which was then coated uniformly onto the surface of a ceramic tube with a pair of gold electrodes with a gap length of about 3 mm and connected by platinum wires. The obtained WO_3 sensing film was vacuum dried at 80 ºC for 2 h. although a Ni-Cr resistor wire was put through the ceramic tube, in all test process, heating voltage still was closed. All the fabricated WO_3 gas sensors were aged at room temperature(RT) for 7 days in air under Blue LED. Six parallel gas sensors were obtained for each powder sample, and the gas sensitivity was measured by identical systems to reduce the operating error. The tested gas was controlled by the auto-gas mixture generator. The gas air-flow was controlled to be 15.0 L/h by the auto-pneumatic valve system and the component ratio of gas was regulated through the device's built-in control system of 4-gas-line. The test chamber

space was approximately 4 mL. The target gases, replacing NO gas-source, were introduced into the test system in various concentrations to assess the response and selectivity of the sensors at RT. The sensors were tested under Blue LED (50W) and the distance between LED and shell of test chamber was kept as about 2 cm. The light intensity on surface of gas sensor could basically maintained at 343~451 mW/cm^2 in all process of gas-sensing measurement. The gas responses to reducing gases and to oxidizing gases were defined as $S = R_a/R_g$ and $S = R_g/R_a$, respectively, where R_a was the resistance of sensor without test gas, and R_g was the resistance of sensor in reducing or oxidizing gas. Short-term stability was evaluated by switching on/off from blank to gas and back to blank in different gas concentrations for a few cycles, whereas the long-term stability was investigated continuously for three months by repeating the short-term measurement under the condition of aging the device at RT. In cycling experiment, when not testing, the gas sensor stored in the aging table at RT in air atmosphere under LED light. Due to the limitation of current equipment, the simulated exhaled-breath was consist of mixture gas (78% N_2 gas, 17% O_2 gas and 5% CO_2 gas) and water vapor, which relative humidity was controlled to be 90 %.

Table S1. Element content of obtained WO_3 sample by XPS measurement.

Sample	Ni/W (mol/mol)*	Sample	Ni/W (mol/mol)*
WO_3	-	1Ni-WO_3	1.1:100
3Ni-WO_3	3.4:100	4Ni-WO_3	4.9:100
5Ni-WO_3	6.1:100	7Ni-WO_3	8.8:100

* this value was calculated from average result (atom/atom) of three random region for same one sample.

Table S2. Gas sensing performance of WO_3 based sensor for NO or NO_2 detection in previous researches and studies.

Materials	Performance and Condition	Reference
Ag_x-WO_3 nanofibers	Operation temperature: 80 ºC; Dark<br>NO_2 10 ppb Response: 10.5;<br>Response/Recovery time: 33 s/ 15 s	[1]
WO_3 nanorods	Operation temperature: 150 ºC; Dark<br>NO 10 ppm Response: 2.29;<br>Response/Recovery time: 82 s/ 112 s	[2]
Pd@Fe_2O_3/ MWCNTs/WO_3	Operation temperature: RT; Dark<br>NO 0.5 ppm Response: 1.18;<br>Response/Recovery time: 291 s/ 511 s	[3]
Pd-WO_3	Operation temperature: 200 ºC; Dark<br>NO 20 ppm Response: 82;<br>Response/Recovery time: 27 s/ 23 s	[4]
Pt/WO_3-CNF	Operation temperature: RT; Dark<br>NO 5 ppm Response: 1.24;<br>Response/Recovery time: 176 s/ 439 s	[5]
WO_3 microtubules	Operation temperature: 50 ºC; Dark<br>NO 1 ppm Response: 74.5;<br>Response/Recovery time: 93 s/ 27 s	[6]
WO_3/V_2CT_x	Operation temperature: RT; Dark<br>NO_2 1 ppm Response: 33.98;<br>Response/Recovery time: 9 s/ 11 s	[7]
WO_3-O_v	Operation temperature: 150 ºC; Dark<br>NO 50 ppb Response: 1.45;<br>Response/Recovery time: - / -	[8]
WO_3 thin film	Operation temperature: 250 ºC; Dark<br>NO 100 ppm Response: 22.7;	[9]

	Response/Recovery time: 172 / 86	
$W_xMo_yO_3$ thin film	Operation temperature: 300 ºC; Dark<br>NO 50 ppm Response: 44.15;<br>Response/Recovery time: 294 / 119	[10]
Ni doped WO_3	Operation temperature: RT; Blue LED<br>NO 50 ppb Response: 5.34;<br>Response/Recovery time: 28 s/ 50 s	This work

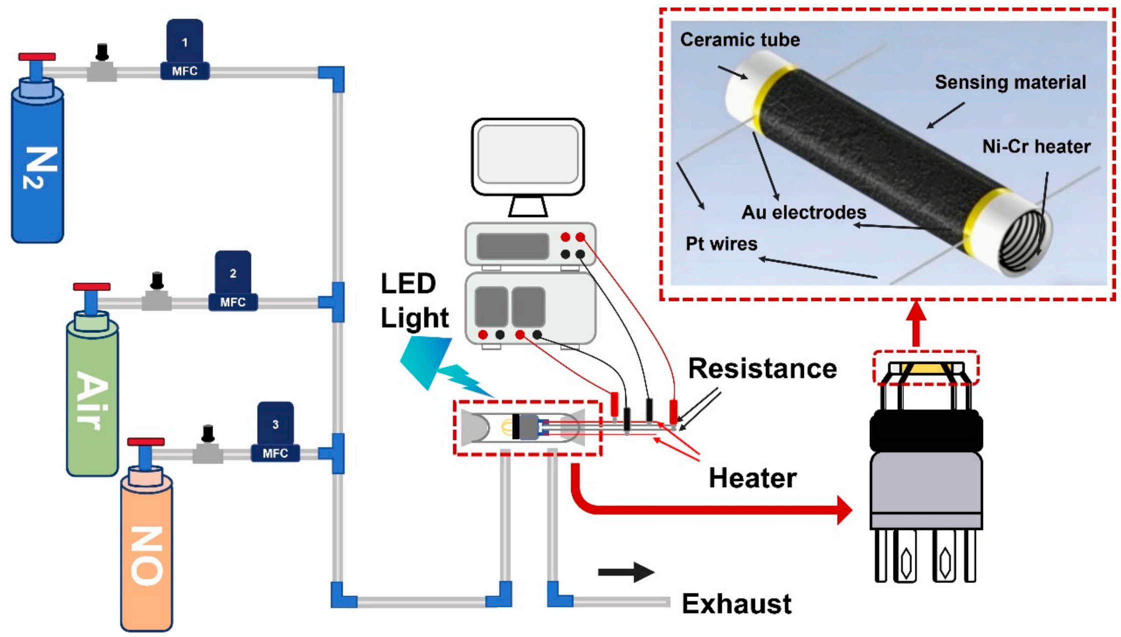

Figure. S1. Schematic diagram of testing system for NO-gas-sensitivity performance.

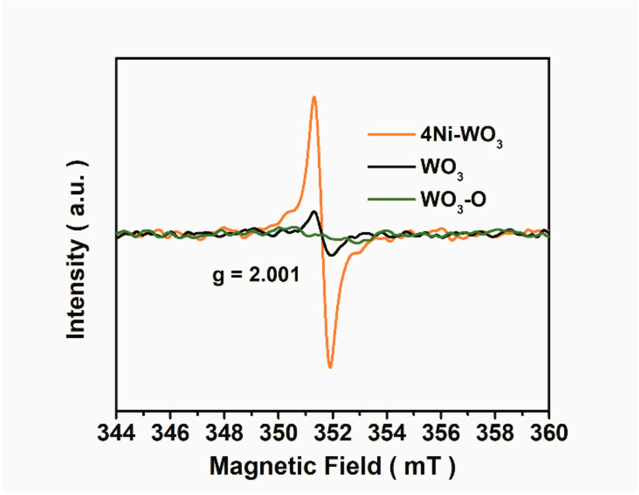

Figure. S2. EPR spectra of $4Ni\text{-}WO_3$, WO_3 and $WO_3\text{-}O$ samples.

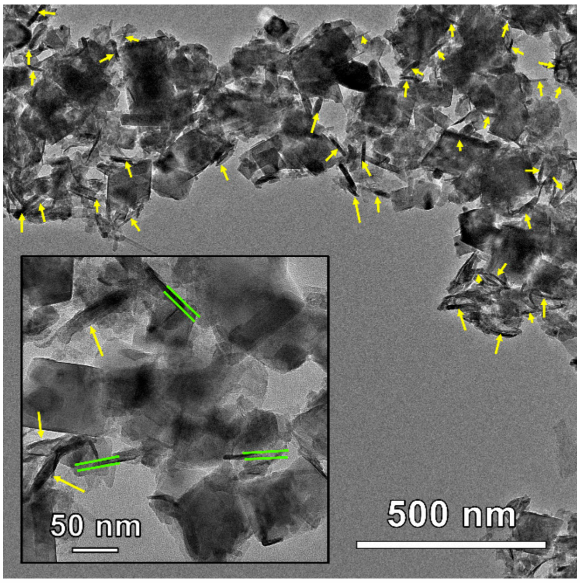

Figure. S3. TEM image of 4Ni-WO_3 sample.

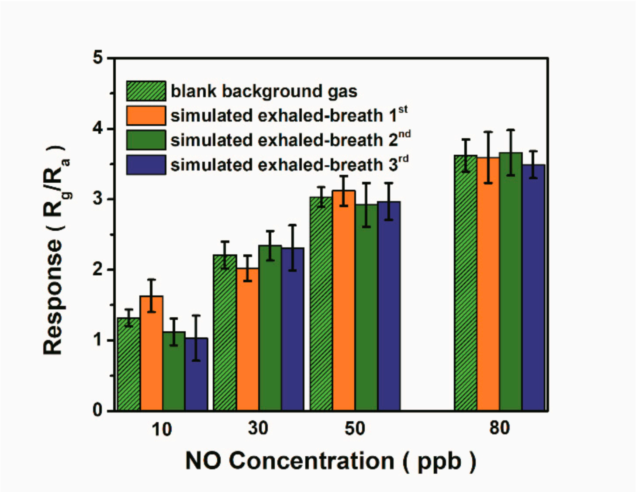

Figure. S4. Gas-sensing performance of 4Ni-WO_3 gas sensor for NO detection in simulated exhaled-breath.

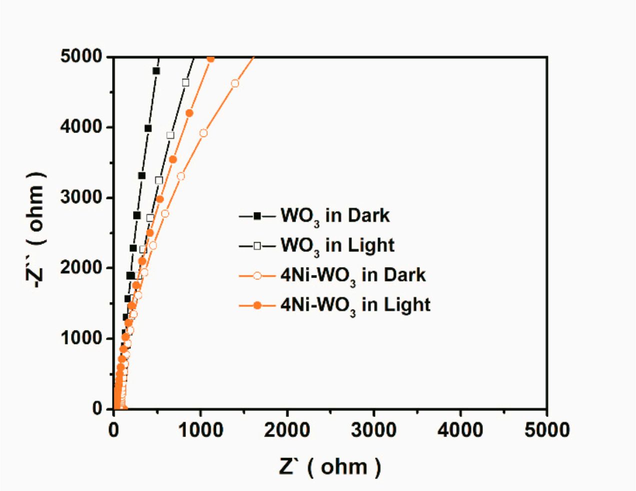

Figure. S5. EIS Nyquist plots of WO_3 and 4Ni-WO_3 sample.

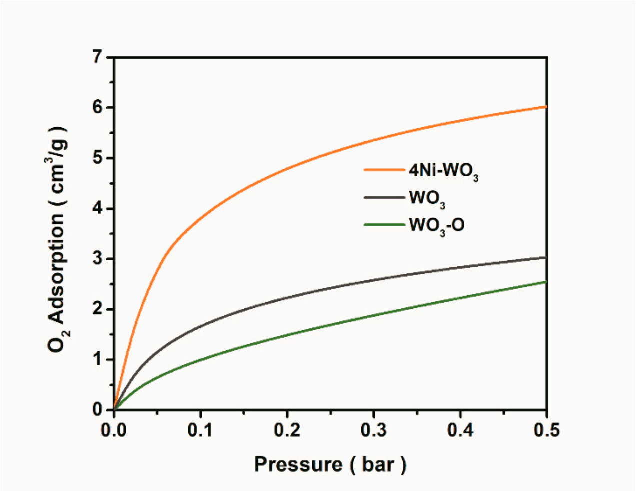

Figure. S6. O_2 adsorption curve of 4Ni-WO_3, WO_3 and WO_3-O samples.

Reference

[1] Zhang, J.; Gao, H.; Leng, D.; Liu, J.; Li, T.; Pan, C.; Yu, B.; Zhang, X.; Guo, J.; Yang, Z.; Zhao, Q.; Gao, J.; Lu, H. Atomic-level tuning of mass-selected Ag clusters for surface functionalization of porous WO_3 nanofibers to enhance NO_2 sensing properties. Chem. Eng. J. 2026, 537, 176649.

[2] Dhall, S.; Prakash, J.; Nigam, A.; Astakala, A.; Sood, K. WO_3-based Chemiresistive sensors for NO detection at low temperatures. Microchem. J. 2025, 215, 114366.

[3] Su, P.G.; Li, M.C. Recognition of binary mixture of NO_2 and NO gases using a chemiresistive sensors array combined with principal component analysis. Sens. Actuators A Phys. 2021, 331, 112980.

[4] Cai, Z.X.; Li, H.Y.; Ding, J.C.; Guo, X. Hierarchical flowerlike WO_3 nanostructures assembled by porous nanoflakes for enhanced NO gassensing. Sens. Actuators B Chem. 2017, 246, 225–234.

[5] Yu, S.; Liu, X.; Wang, J.; Li, Q.; Pang, Y.; Zhang, L.; Yang, C.; Meng, Q.; Wang, C.; Jing, Q.; Chen, J.; Liu, B. A Room-temperature, high-ppb-level NO gas sensor based on Pt/WO_3 co-decorated carbon nanofibers towards asthma-relevant breath analysis application. Sensors 2026, 26, 1069.

[6] Wu, Z.-J.; Chen, G.-L.; Li, Q.-S.; Lv, W.-C.; Xin, J.-J.; Song, K. WO_3 microtubules supported by nanoparticles to construct high response and low-temperature nitric oxide sensor from discarded cotton towels. Microchim. Acta 2026, 193, 454.

[7] Bai, H.; Guo, R.; Zhou, Y.; Feng, C.; Chen. Y.; Zhang, S.; Feng, Y.; Liu, W.; Liu, K.; Guo, F.; Wang, J.; Chen, D.; Zhang, R.; Zheng, Y. A room-temperature wireless NO_2 gas sensor enabled by WO_3 modified V_2CT_x nanosheets. Chem. Eng. J. 2026, 534, 174763.

[8] Chen, R.; Si, R.; Dai, Q.; Yang, L.; Hou, M.; He, S.; Li, X.; Zhang, S.; Xiang, L.; Guo, S.; Xia, Y. Oxygen vacancy-engineered WO_3 sensors with chemoselective gating for ultralow NO breathomics: Towards point-of-care asthma diagnostics. Chem. Eng. J. 2026, 533, 174759.

[9] Singh, S.; Gurawal, P.; Malik, G.; Adalati, R.; Kaur, D.; Chandra, R.; Highly responsive and selective NO gas sensing based on room temperature sputtered nanocrystalline WO_3/Si thin films. Micro Nanostruct. 2024, 188, 207794.

[10] Singh, S.; Adalati. R.; Gurawal, P.; Devi, R.; Malik, G.; Kaur, D.; Chandra, R. Nano-interfaced $W_xMo_yO_3$ heterostructure based highly selective and sensitive NO gas sensor. Sens. Actuators, B 2026, 451, 139436.
